# Filamentation and inhibition of prokaryotic CTP synthase with ligands

**DOI:** 10.1002/mlf2.12119

**Published:** 2024-05-02

**Authors:** Chenjun Guo, Zixuan Wang, Ji‐Long Liu

**Affiliations:** ^1^ School of Life Science and Technology ShanghaiTech University Shanghai China; ^2^ Department of Physiology, Anatomy and Genetics University of Oxford Oxford UK; ^3^ Shanghai Clinical Research and Trial Center Shanghai China

**Keywords:** CTP synthase, Cryo‐electron microscopy, cytoophidium, metabolic filament, species specific inhibition

## Abstract

Cytidine triphosphate synthase (CTPS) plays a pivotal role in the de novo synthesis of cytidine triphosphate (CTP), a fundamental building block for RNA and DNA that is essential for life. CTPS is capable of directly binding to all four nucleotide triphosphates: adenine triphosphate, uridine triphosphate, CTP, and guanidine triphosphate. Furthermore, CTPS can form cytoophidia in vivo and metabolic filaments in vitro, undergoing regulation at multiple levels. CTPS is considered a potential therapeutic target for combating invasions or infections by viral or prokaryotic pathogens. Utilizing cryo‐electron microscopy, we determined the structure of *Escherichia coli* CTPS (ecCTPS) filament in complex with CTP, nicotinamide adenine dinucleotide (NADH), and the covalent inhibitor 6‐diazo‐5‐oxo‐ l‐norleucine (DON), achieving a resolution of 2.9 Å. We constructed a phylogenetic tree based on differences in filament‐forming interfaces and designed a variant to validate our hypothesis, providing an evolutionary perspective on CTPS filament formation. Our computational analysis revealed a solvent‐accessible ammonia tunnel upon DON binding. Through comparative structural analysis, we discern a distinct mode of CTP binding of ecCTPS that differs from eukaryotic counterparts. Combining biochemical assays and structural analysis, we determined and validated the synergistic inhibitory effects of CTP with NADH or adenine on CTPS. Our results expand our comprehension of the diverse regulatory aspects of CTPS and lay a foundation for the design of specific inhibitors targeting prokaryotic CTPS.

## Impact statement

Cytidine triphosphate synthase (CTPS) is pivotal for life, as it catalyzes the essential de novo synthesis of cytidine triphosphate (CTP), a fundamental building block for DNA and RNA. By decoding the intricate structure of *Escherichia coli* CTPS and its regulatory interactions, we shed light on targeted drug design against prokaryotic infections. This research not only enriches our understanding of CTPS but also provides insights into the evolution of metabolic filaments across prokaryotic and eukaryotic species.

## INTRODUCTION

Cytidine triphosphate synthase (CTPS) plays a critical role in catalyzing the final and rate‐limiting step of de novo cytidine triphosphate (CTP) synthesis. CTPS is composed of a glutamine amidotransferase (GAT) domain and a kinase‐like ammonia ligase (AL) domain^1^, ^2^. It utilizes ammonia generated from glutamine and adenine triphosphate (ATP) as an energy source to convert the substrate uridine triphosphate (UTP) into CTP^3^. Considering the indispensability of its product CTP in DNA, RNA, and phospholipid synthesis, CTPS is meticulously regulated through a variety of mechanisms^4^, ^5^, ^6^, ^7^, ^8^, ^9^, ^10^, ^11^. While CTP can inhibit the activity of CTPS, guanidine triphosphate (GTP) serves as an enzymatic regulator, stimulating the reaction at low concentrations and inhibiting it at higher levels^12^. The redox cofactor nicotinamide adenine dinucleotide (NADH) has been found to inhibit *Escherichia coli* CTPS (ecCTPS), holding potential physiological relevance^13^.

In solution, ecCTPS exists in an inactive dimeric form under dilute conditions. Upon the addition of the substrates UTP and ATP, it transitions into an active tetrameric form, which is also observed in eukaryotic CTPS^14^. The presence of product CTP prompts CTPS to shift into an inactive tetrameric state for both prokaryotic and eukaryotic enzymes^15^. Recent studies have revealed that CTP can bind to CTPS at two distinct binding sites for various eukaryotic species^16^, ^17^, ^18^.

Beyond oligomerization, CTPS demonstrates the capability to further aggregate and form metabolic filaments^16^, ^19^, ^20^, ^21^, ^22^, ^23^. Filaments of CTPS have been confirmed and characterized in multiple species and conditions, where ecCTPS has been shown to form large‐scale inhibitory filaments in the presence of product CTP^20^. Both human CTPS1 and CTPS2 (hCTPS1 and hCTPS2), along with *Drosophila* CTPS (dmCTPS), can form different filaments under the conditions of ATP, UTP, or CTP, which have varying effects on reaction promotion. Ura7 and ura8 from budding yeast also form filaments under substrate and product conditions and respond to changes in pH^16^.

CTPS has been observed to aggregate into micron‐scale nonmembrane‐bound organelles named cytoophidium (plural cytoophidia) due to their snake‐like appearance^19^, ^24^, ^25^. Cytoophidia are highly conserved across multiple species and all three domains of life, suggesting their special biological role. In prokaryotes, cytoophidia were initially observed and demonstrated to be crucial for the morphology of bacteria, such as *Caulobacter crescentus* ^19^. *Drosophila* have served as a classic model for studying CTPS cytoophidia^26^. Through experiments involving point mutations and functional analysis, CTPS cytoophidia have been found to have close ties to adipose tissue architecture^27^.

Despite its complex regulation, the vital biological role of CTPS positions it as a potential drug target for diseases, such as cancer^28^, bacterial infections^7^, and parasitic infections^29^. As our understanding deepens, the design for the targeted regulation of CTPS and species‐specific regulation is becoming more feasible. Recently, McLeod et al. unveiled the inhibitory effects of gemcitabine‐5′‐triphosphate, a therapeutic agent against solid tumors, on ecCTPS^30^. It exhibited a strong binding affinity to CTPS, approximately 80‐fold greater than CTP, providing novel insights into inhibitor development^31^. Utilizing cryo‐electron microscopy (cryo‐EM), Lynch et al. revealed the mechanisms of selective inhibition of human CTPS and laid a foundation for the design of immunosuppressive therapies^17^.

In this study, we elucidated the structure of ecCTPS in complex with CTP, NADH, and the covalent inhibitor 6‐diazo‐5‐oxo‐l‐norleucine (DON). Incorporating phylogenetic analysis and bioengineering, we gained evolutionary insights into CTPS filament formation. Through computational analysis, we revealed a solvent‐accessible ammonia tunnel upon DON binding. Our structures captured the interaction between NADH and ecCTPS. In addition, biochemical assays revealed that NADH achieved synergistic inhibition by interacting with the ATP binding pocket through its adenine portion. Furthermore, we identified distinct CTP binding and inhibition modes in ecCTPS compared to already‐resolved eukaryotic CTPS structures due to sequence variations. Our findings lay the groundwork for developing species‐specific CTPS inhibitors.

## RESULTS

### Structure of ecCTPS bound with CTP, NADH, and DON

Previous studies have revealed that CTPS possesses the intriguing ability to spontaneously assemble into filaments in vitro under different conditions^19^. Structures of CTPS filament have been determined in various eukaryotic organisms, including humans, *Drosophila*, and budding yeast. Previously, researchers observed and characterized CTPS filaments from prokaryotes in vitro and in vivo.

**Figure 1:**
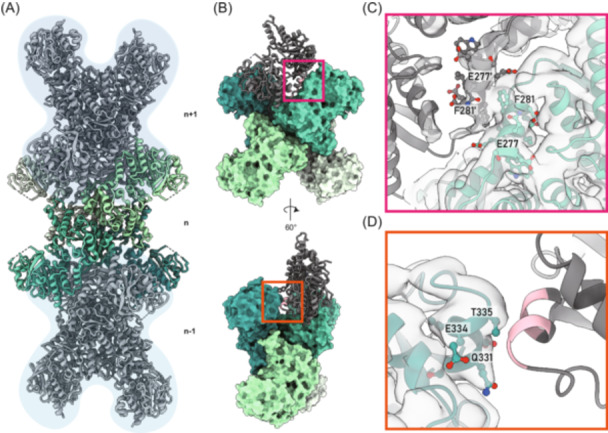
Assembly of ecCTPS filament. (A) Structure of the ecCTPS filament. The model is composed of three helical units. The central ecCTPS tetramer is colored by different protomers. The upper and lower layers of the tetramer are depicted using gray ribbons with a light‐blue background. (B) Contact sites between adjacent tetramers. One ecCTPS tetramer is shown by the solvent‐accessible surface, while another protomer adjacent to ecCTPS is depicted using a gray ribbon. Amino acids within a 4.5 Å distance are highlighted in pink. Two different angled views are presented. (C, D) Major assembly interfaces of the ecCTPS filament. Zoomed‐in views of the magenta and orange boxes are shown in (B). The map density is displayed as a transparent white surface. CTPS, cytidine triphosphate synthase; ecCTPS, *Escherichia coli* CTPS.

To establish a more robust structural foundation for the study of prokaryotic CTPS filaments, we employed a recombinant expression approach to purify and isolate the CTPS protein from *Escherichia coli* (Figure S1). Utilizing cryo‐EM and single particle analysis, we resolved the structure of purified ecCTPS bounded with CTP, NADH, and DON, achieving a resolution of 2.9 Å (Figures 1A and S2–4). During the sample preparation process, under conditions with product CTP, we observed a distinct filamentous arrangement of ecCTPS in the cryo samples (Figure S3). The results of the two‐dimensional (2D) classification provided a clear visualization of the ecCTPS filament, wherein it assembles into filaments with CTPS tetramers as the helical unit (Figure S3).

Consistent with the findings of Barry et al., the interface of the ecCTPS filament comprises two major interaction regions (Figure 1B). One of these regions is located in the linker domain of CTPS, where chains A and A′ engage in a symmetric complementary way (Figure 1C). Notably, residue F281 appears to form Van der Waals interactions with F281′. These interactions likely contribute to the stability of the ecCTPS filament. The second interface is situated between helices 330–335 within the GAT domain (Figure 1D).

### Evolution of CTPS filament assembly

**Figure 2:**
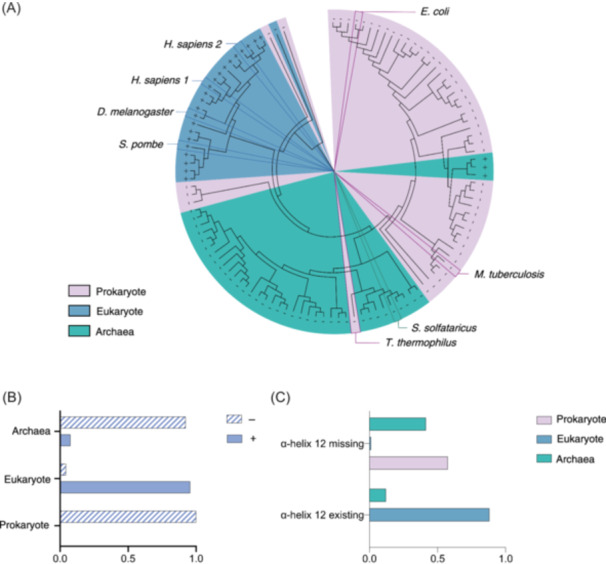
Phylogenetic analysis of CTPS. (A) Phylogenetic tree of CTPS consisting of 112 CTPS sequences. “+” represents the existence of helix 12, and “−” represents the lack of helix 12. Eukaryotes, prokaryotes, and archaea are distinguished by different colors. (B) Classification by eukaryotes, prokaryotes, and archaea. This histogram indicates the ratios of the existence or absence of helix 12 in eukaryotes, prokaryotes, and archaea. (C) Classification based on the existence or absence of helix 12. The ratios of eukaryotes, prokaryotes, and archaea in the helix 12 existing and missing conditions are shown, respectively.

In comparison to eukaryotic CTPS, the assembly interactions of ecCTPS filament exhibit significant distinctions. The assembly interface of solved eukaryotic CTPS filaments is primarily centered on helix 12, a sequence that is absent in the *E. coli* counterpart (Figure S5). To systematically analyze this phenomenon from an evolutionary perspective, we selected CTPS sequences from 112 species to construct a phylogenetic tree. This data set included 50 prokaryotes, 23 eukaryotes, and 39 archaea. The presence or absence of helix 12 was used as a classification criterion (Figures 2A and S6).

The evolutionary analysis revealed that among the 112 selected species, helix 12 is present in nearly all eukaryotes (>95%) and absent in all prokaryotes and the majority of archaea (92%) (Figure 2B). Based on the presence or absence of helix 12 for sequence classification, we observed that the majority of organisms possessing helix 12 were eukaryotes, with only a small fraction belonging to archaea. Conversely, among the organisms lacking helix 12, prokaryotes and archaea constituted the predominant groups. Notably, within the analyzed data set, only one eukaryotic organism was found to be devoid of helix 12 (Figure 2C).

The insertion of helix 12 into the GAT domain allows ecCTPS to form filaments with substrates UTP and ATP. Based on both previous research and our experimental findings, it has been established that the wild‐type ecCTPS is capable of forming filaments in the presence of product CTP. However, filament formation was not observed in the presence of substrates UTP and ATP^20^.

To elucidate the necessity and role of helix 12 in CTPS filament formation, we engineered, expressed, and purified ecCTPS with an inserted hCTPS helix 12 (referred to as ecCTPS^helix12+^) (Figure S7).

**Figure 3:**
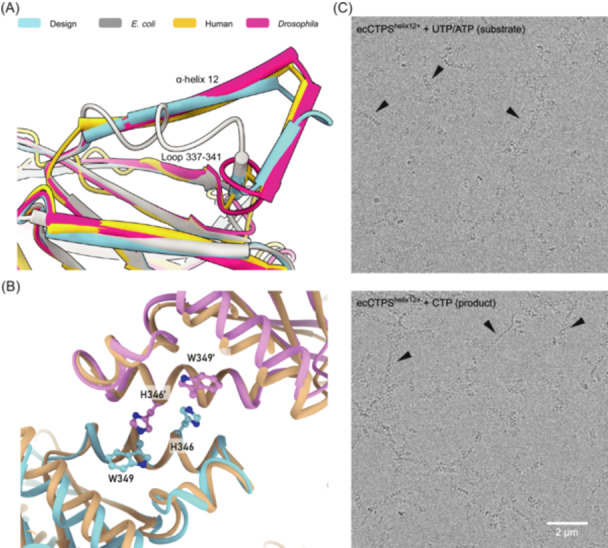
Properties of ecCTPS^helix12+^. (A) Comparison of the helical assembly interface between ecCTPS^helix12+^ and other representative structures. The structure of ecCTPS^helix12+^ is predicted by AlphaFold2. Models are colored according to the above color key. (B) Simulated helical interface of ecCTPS^helix12+^. Two monomers of ecCTPS^helix12+^ are aligned to the structure of the hCTPS1 filament by helix 12. The model of hCTPS1 is colored in champagne. (C) Cryo‐electron micrograph of ecCTPS^helix12+^ under substrate and product conditions. Filaments are indicated by black arrows. The scale bar is 2 μm.

We employed AlphaFold2 to predict the structure of ecCTPS^helix12+^. Among the five structures predicted, rank 2 exhibited the best performance across interchain predicted alignment error and predicted local distance difference test, leading us to select this model for subsequent analysis (Figure S8). Structural alignment revealed that ecCTPS^helix12+^ possesses a markedly different secondary structure compared to wild‐type ecCTPS. The insertion of the human CTPS sequence resulted in the extension of the originally shorter loop 337–341, forming a complete helix that resembles α‐helix 12, which is responsible for filament assembly in eukaryotic CTPS (Figure 3A). When we aligned the predicted model with hCTPS1 filament based on the filament assembly interface, we observed a high degree of similarity, with a root‐mean‐squared deviation (RMSD) of 0.479 Å. H346 and W349 exhibited patterns consistent with H355 and W358 of hCTPS1, which play vital roles in the filament assembly (Figure 3B). This implies that ecCTPS^helix12+^ possesses a structural basis for assembling into filaments that resemble those in hCTPS1.

To further investigate the nature of ecCTPS^helix12+^, we prepared samples of it in both substrate and product states. Upon analyzing cryo samples of ecCTPS^helix12+^, we observed that this engineered variant not only formed filaments in the presence of product CTP but also exhibited filament formation in the presence of substrates UTP and ATP (Figure 3C). Intriguingly, the arrangement of the formed filaments exhibited a connected X‐shaped pattern, resembling the previously observed helix 12–12–mediated helical interface in eukaryotic CTPS filaments^20^. This result demonstrates that the insertion of hCTPS helix 12 is sufficient to alter the assembly mode of ecCTPS filaments, suggesting the pivotal role of helix 12 in determining the CTPS filament assembly pattern.

Our study established a connection between protein sequences and the assembly of CTPS filaments while also providing an evolutionary perspective on the formation of CTPS filaments.

### DON binding and ammonia tunnel formation of ecCTPS

CTPS is composed of GAT and AL domains, and during the reaction, the intermediate product ammonia is transferred between the two domains through an internal ammonia tunnel. DON is an irreversible inhibitor of CTPS that can covalently bind to CTPS and inhibit its activity.

**Figure 4:**
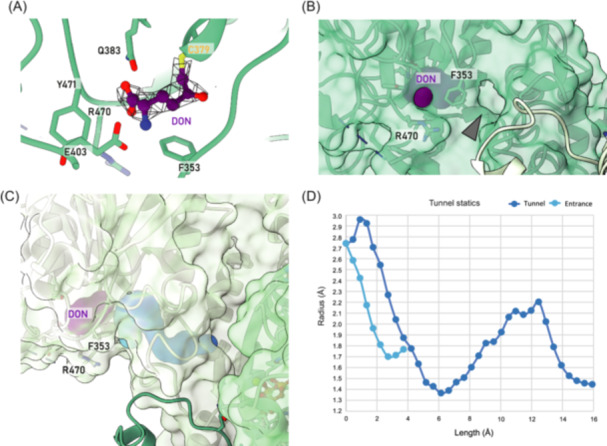
Ammonia tunnel of 6‐diazo‐5‐oxo‐l‐norleucine (DON) inhibited ecCTPS. (A) Binding of DON to ecCTPS. DON is represented by a purple ball‐and‐stick model, and the density map is depicted as a mesh. (B) Ammonia entrance during DON inhibition. The surface of ecCTPS is depicted in transparent green, while the surface of DON is shown in purple. Gray arrows indicate the location of the entrance. (C) Ammonia tunnel of DON‐inhibited ecCTPS. The surface of ecCTPS is transparent, with the entrance and ammonia tunnel represented in light blue and deep blue, respectively. (D) Tunnel statics. The two curves in light blue and deep blue correspond to the entrance and tunnel parameters in panel (C), respectively. The *x*‐axis represents the distance along the channel center, while the *y*‐axis represents the maximum accessible radius from the corresponding channel center, measured in angstroms.

In 1971, Levitzki et al. demonstrated that even after DON binding, prokaryotic CTPS can still use NH_4_^+^ as a substrate for CTP synthesis^32^. In our obtained structure, DON forms a covalent bond with C379, a residue that is critical for the catalytic function of ecCTPS. Surrounding amino acids, such as F353, contribute to the binding and stabilization (Figure 4A). Upon the conjunction of DON, the GAT domain adopts a closed conformation, preventing external NH_4_^+^ from accessing the ammonia tunnel through the catalytic pocket of GAT for further utilization by the AL domain (Figure 4B).

Through computational analysis, we observed the presence of a solvent‐accessible channel on the side of F353 close to the AL domain, connecting an internal cavity within the GAT domain (Figure 4B,C). The minimum diameter of this channel exceeded 1.7 Å, allowing the passage of NH_3_ or NH_4_^+^ (Figure 4D). This cavity is also positioned above the ammonia tunnel that connects to the AL domain, where we identified a minimum diameter of the ammonia tunnel above 1.3 Å, allowing the transfer of ammonia. The existence of this entrance to the ammonia tunnel explains the feasibility of ecCTPS utilizing NH_4_^+^ as a substrate when DON is bound to the GAT pocket.

### Diverse binding modes of CTP in CTPS

The product CTP serves as a natural inhibitor of various eukaryotic CTPSs and can bind to the substrate binding pockets of UTP or ATP in two distinct inhibitory modes: competitive and noncompetitive with substrate UTP^17^, ^18^.

**Figure 5:**
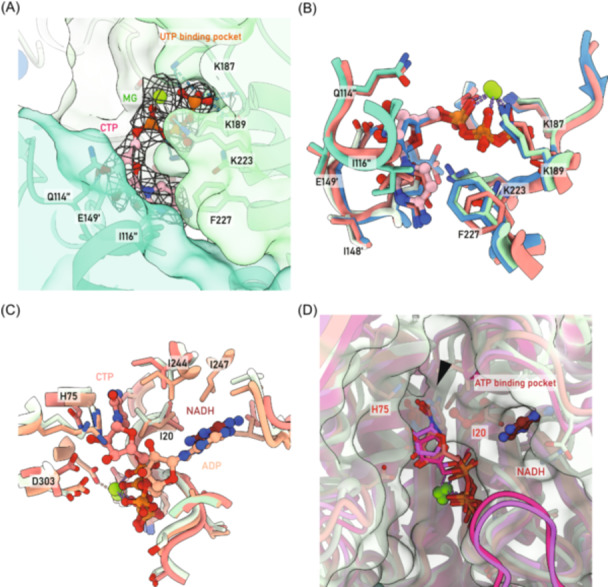
Diversities of cytidine triphosphate (CTP) binding pockets among organisms. (A) Classical CTP binding pocket. CTP is represented by pink sphere‐and‐stick models, ecCTPS is color‐coded by protomers, map density is depicted as a mesh, and hydrogen bonds are shown as blue dashed lines. MG, magnesium ions represented by green spheres. (B) Comparison of classical CTP binding sites. Models of *Drosophila* cytidine triphosphate synthase (dmCTPS), ecCTPS (by X‐ray), and ecCTPS (by EM) are colored in red, blue, and green, respectively, with the corresponding Protein Data Bank (PDB) codes 7DPW, 5TKV, and 8I9O. (C) Comparisons of nonclassical CTP binding sites. Models of dmCTPS with CTP binding in both classical and nonclassical pockets, ecCTPS with ADP, CTP binding, and the model of this study are displayed, with the PDB codes 7DPW, 2AD5, and 8I9O. They are colored red, beige, and green, respectively. (D) Comparison of allosteric inhibitory sites for CTP binding. The clash of nonclassical CTP binding with the simulated solvent‐accessible surface of ecCTPS is indicated by a black arrow. Pink, reddish, and magenta ribbons represent models of dmCTPS, hCTPS1, and hCTPS2, respectively, with the PDB codes 7DPW, 7MH0, and 7MH1. Structures are aligned by fitting them into the map of ecCTPS.

In our structure of ecCTPS, CTP binding was observed in the UTP binding pocket (Figure 5A), consistent with previously resolved ecCTPS–CTP complexes^18^, ^21^ (Figure 5B). CTP shares the triphosphate binding site with UTP, and interactions between coenzyme magnesium ions and the triphosphate portion further stabilize CTP binding. The cytidine portion and ribose of CTP are enveloped by three adjacent protomers, which are stabilized via electronic interactions (Figure 5A,B).

Previous research has revealed that product CTP can bind to CTPS near the substrate ATP binding pocket. This binding mode, initially discovered in dmCTPS in 2021, emerged years after the classical CTP binding site was identified^18^. As a result, this unique CTP binding mode was coined the “nonclassical binding of CTP”. Subsequently, the existence of the nonclassical CTP binding mode was also confirmed in both hCTPS1 and hCTPS2^17^.

In this nonclassical binding mode, the triphosphate portion of CTP shares the same pocket as ATP, while the cytidine portion of CTP adopts a distinct binding mode alongside the adenine portion of ATP (Figure 5C). However, we did not observe the density of CTP in proximity to the ATP binding pocket in our map. Aligning the structure with those of dmCTPS and hCTPS containing nonclassical CTP binding, we identified a clash between the CTP portion of the eukaryotic CTPS model and the solvent‐accessible surface of the ecCTPS model due to hindrance by amino acids H75 and I20 (Figure 5D). This clash impedes CTP from binding to ecCTPS in a noncompetitive inhibitory mode similar to that in eukaryotic CTPS within the ATP pocket.

### NADH binds to ecCTPS in the ATP binding pocket

**Figure 6:**
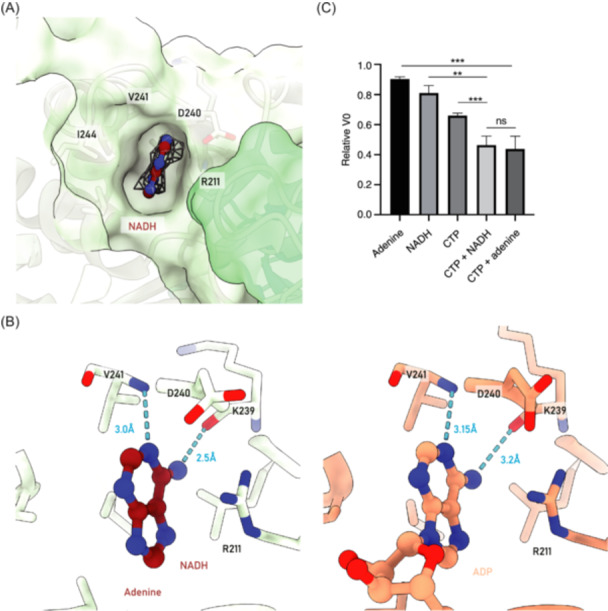
Nicotinamide adenine dinucleotide (NADH)‐specific binding and inhibition of ecCTPS. (A) Density of the adenine portion of NADH. Map density is depicted as a mesh. (B) Comparison of NADH and ADP binding. Hydrogen bonds are indicated by blue dashes. Left: model of this study. Right: PDB 2AD5. (C) Enzyme activity assays for inhibition by CTP, NADH, and adenine on ecCTPS. The initial velocity (V0) in the absence of any inhibitor is the benchmark. The relative V0 in the presence of CTP, CTP and NADH, and CTP and adenine are 0.66, 0.46, and 0.44, respectively. The concentrations of CTP, NADH, and adenine were 0.4, 2, and 2 mM, respectively.

In earlier research, Habrian et al. discovered that NADH can synergistically inhibit CTPS along with CTP^13^. However, a molecular basis for this regulation was not provided. We observed NADH binding in the ATP binding pocket in our model (Figure 5D). At the 1σ counter level, the adenine portion of NADH displayed clear and distinct density, while the remaining portion lacked stable density (Figure 6A).

Within the ATP binding pocket, the adenine portion of NADH exhibits a mode of interaction similar to that of ADP with the ecCTPS. The N1 atom forms a set of hydrogen bonds with the nitrogen atom on the V241 backbone, with a distance of 3.0 Å. Additionally, the N6 atom forms another set of stronger hydrogen bonds with the oxygen atom on the K239 backbone, with a distance of 2.5 Å. Furthermore, adenine is within 3.8 Å of the plane of the R211 side chain, forming a strong pi–pi interaction, which, along with surrounding hydrophobic amino acids, stabilizes the binding of adenine (Figure 6B). According to our model, the adenine portion of NADH exhibits a stronger interaction with the ATP binding pocket of ecCTPS compared to the adenine portion of ADP (Figure 6B). This could be attributed to the failure of NADH's remaining functional group to achieve stable binding, thereby providing the adenine portion of NADH with ample space to optimize its binding. This also suggests that NADH's interaction with ecCTPS may depend on the adenine portion, accounting for the relatively weak inhibition of ecCTPS reported by Habrian et al.^13^

### Synergistic inhibition of ecCTPS by CTP with NADH or adenine

To validate the binding effect of NADH and its regulatory role on ecCTPS, we examined the inhibitory effects of both NADH and adenine in combination with CTP on CTPS activity (Figure 6C). In the control setup, CTP alone significantly inhibited CTPS activity. The presence of NADH further suppressed the reaction, while the combination of adenine and CTP exhibited a similar degree of inhibition to that of the combination of CTP and NADH. Enzyme activity assays confirmed the synergistic inhibition of CTPS activity by NADH and CTP and indicated that adenine also possesses this capability.

Our structure provides a structural foundation for the synergistic inhibition of ecCTPS by NADH and CTP. Furthermore, through enzyme activity assays, we propose that NADH's inhibition of CTPS is likely dependent on the affinity between the adenine portion and the ATP pocket of ecCTPS.

## DISCUSSION

ecCTP synthase is a classical case in the study of CTPS and protein regulation. Numerous biochemical characteristics and regulatory patterns of CTPS have been revealed by ecCTPS in previous studies. In 2010, it was discovered that ecCTPS could form filaments in vitro, and subsequently, the structure was resolved^19^, ^20^. However, the limited resolution hindered a more in‐depth understanding. The difference in its formation pattern compared to eukaryotic CTPS also lacks a reasonable explanation^21^. In 2016, it was found that NADH could regulate the activity of CTPS, but the mechanism remained unclear^13^.

In this study, we determined the structure of the complex formed by ecCTPS with CTP, DON, and NADH, shedding light on the interaction pattern between CTPS and the alternative nucleotide ligand NADH. Moreover, our findings refine our comprehension of the regulation exerted by classical ligands, such as CTP and DON, on CTPS. The near‐atomic resolution structures provide robust support to and validation for our conclusions.

Despite these advances, there are some limitations in our research stemming from experimental constraints. We based our criteria for evolutionary analysis solely on the presence or absence of helix 12. However, it is important to note that other differences could potentially influence filament formation, such as the nonconserved helix 18 at the C‐terminal, which appears to have a possible connection to evolutionary relationships. Furthermore, the engineered ecCTPS, successfully assembled into filaments with substrates, displayed certain morphological similarities to eukaryotes. Further investigation is required to determine whether the assembly mode and regulation pattern for the engineered ecCTPS are identical with the eukaryotic CTPS.

Nevertheless, our phylogenetic analysis and bioengineering offer valuable insights. Specifically, they raise intriguing questions about how the two types of filaments, under the product state and the substrate state, respectively, evolved. Our findings may imply an asynchronous evolution, prompting us to delve deeper into the significance of these distinct filamentation events in vivo.

## MATERIALS AND METHODS

### ecCTPS plasmid construction and protein purification

The full‐length *ecCTPS* gene was cloned into a pET28a vector with a C‐terminal 6× His tag and transformed into *E. coli* Transetta (DE3) cells for expression. Transformed cells were at first cultured in 6–8 ml LB medium at 37°C with 220 rpm for about 12 h and then transferred to 1 l LB medium. When its OD_600_ reached a range of 0.6–0.8, 0.1 mM isopropyl β‐d‐1‐thiogalactopyranoside was added for induction. Cells were pelleted by centrifugation at 4500 rpm for 10 min followed by resuspension in cold lysis buffer (50 mM Tris‐HCl pH 7.5, 500 mM NaCl, 10% glycerol, 20 mM imidazole, 1 mM PMSF, 5 mM β‐mercaptoethanol, 5 mM benzamidine, 2 μg/ml leupeptin, and 2 μg/ml pepstatin). The mixed suspension of bacteria and lysis buffer was disrupted by high pressure at 800 bar and centrifuged at 16,000*g* for 30 min at 4°C to collect supernatant, which was then incubated with equilibrated Ni‐Agarose (Qiagen) for 1.5 h. Next, Ni‐Agarose was washed by washing buffer (50 mM Tris‐HCl pH 7.5, 500 mM NaCl, 10% glycerol, 40 mM imidazole, 5 mM β‐mercaptoethanol), and proteins were eluted with elution buffer (30 mM Tris‐HCl pH 7.5, 100 mM NaCl, 240 mM imidazole). SuperoseTM 6 Increase 10/30 GL column and AKTA Pure (Cytiva) were used for further purification of peak fractions. Finally, CTPS was eluted with buffer containing 100 mM NaCl and 30 mM Tris‐HCl pH 7.5 and stored at −80°C for further use.

### CTPS activity assay

Purified CTPS protein was incubated in the reaction buffer containing 100 mM NaCl, 30 mM Tris‐HCl pH 7.5, 1 mM ATP, 1 mM UTP, 0.2 mM GTP, and 10 mM MgCl_2_ for 15 min at 37°C. To initiate the reaction, 10 mM prewarmed glutamine was added to the mixture. For inhibitor assay, 0.4 mM CTP and 2 mM NADH or adenine were added to the mixture before incubation. Absorption of a wavelength of 291 nm of each reaction mixture was measured using SpectraMax i3 as the indication for CTP production at individual time points^33^, and measurements were made at 10 or 15 s intervals until the absorption value tended to be constant.

### Phylogenetic tree construction

A total of 112 sequences of CTPS of different organisms were acquired from the Swiss‐Prot database^34^. A phylogenetic tree was built by Unweighted Pair Group Method (UPGMA)^35^ using MEGA11^36^. Poisson regression method was chosen for the modeling and pairwise deletion was applied for gaps treatment. MUSCLE^37^ program was used for alignment of the sequence of *Homo sapiens* CTPS1 (UniProtKB:P17812) and CTPS2 (UniProtKB:Q9NRF8), *Schizosaccharomyces pombe* CTPS (UniProtKB:O42644), *Drosophila melanogaster* CTPS (UniProtKB:Q9VUL1), *E. coli* CTPS (UniProtKB:P0A7E5), *Mycobacterium tuberculosis* CTPS (UniProtKB:P9WHK7), *Thermus thermophilus* CTPS (UniProtKB:Q5SIA8), and *Sulfolobus solfataricus* CTPS (UniProtKB:Q980S6). The result of sequence alignment was visualized by ESPript 3^38^ which rendered sequence similarities and structure information taking the crystal structure of the tetrameric form of human CTPS1 (PDB EntryID:7MGZ) as a reference.

### Design and structure prediction of engineered protein

ecCTPS^helix12+^ was designed by inserting helix 12 from hCTPS1 into ecCTPS after E399. The structure of ecCTPS^helix12+^ was predicted using ColabFold^39^, with the protein sequence “MTTNYIFVTGGVVSSLGKGIAAASLAAILEARGLNVTIMKLDPYINVDPGTMSPIQHGEVFVTEDGAETDLDLGHYERFIRTKMSRRNNFTTGRIYSDVLRKERRGDYLGATVQVIPHITNAIKERVLEGGEGHDVVLVEIGGTVGDIESLPFLEAIRQMAVEIGREHTLFMHLTLVPYMAASGEVKTKPTQHSVKELLSIGIQPDILICRSDRAVPANERAKIALFCNVPEKAVISLKDVDSIYKIPGLLKSQGLDDYICKRFSLNCPEANLSEWEQVIFEEANPVSEVTIGMVGKYIELPDAYKSVIEALKHGGLKNRVSVNIKLIDSQDVETRGVEEEPVRYHEAWQILKGLDAILVPGGFGYRGVEGMITTARFARENNIPYLGICLGMQVALIDYARHVANMENANSTEFVPDCKYPVVALITEWRDENGNVEVRSEKSDLGGTMRLGAQQCQLVDDSLVRQLYNAPTIVERHRHRYEVNNMLLKQIEDAGLRVAGRSGDDQLVEIIEVPNHPWFVACQFHPEFTSTPRDGHPLFAGFVKAASEFQKRQAKHHHHHH” serving as the input. Among the five prediction results, the model of rank2 exhibited the best performance and was selected for the subsequent analysis.

### Cryo‐EM grid preparation and data collection

For preparing the ecCTPS filament sample, CTPS was incubated with 0.58 mM DON, 1 mM CTP, 0.5 mM NADH, and 10 mM MgCl_2_ at a final concentration of 7 μM for 30 min at 0°C. Samples were prepared with 300 holey golden film (M01Au300‐R1.2/1.3) and FEI Vitrobot (4°C temperature, multiple rounds of sample application and blotting before vitrification, 3.5 s blotting time, −1 blot force). Images were taken with a Gatan K3 summit camera on a FEI Titan Krios electron microscope operated at 300 kV. The magnification was 22,500× in superresolution mode with the defocus range of −1.2 to −1.8 μm and a pixel size of 1.06 Å. The total dose was 50e−/Å^2^ subdivided into 40 frames at 2.8s exposure using SerialEM.

### Image processing

We used MotionCor2 for alignment of the raw movie frames, and CTFFIND4 for contrast transfer function (CTF) estimation. A total of 2760 images were selected for subsequent analysis. By employing template‐free particle picking in Relion, we obtained coordinates for 3,401,539 particles. The single particle analysis strategy was carried out during the remaining processes. Particles were downsampled to a pixel size of 2.12 Å for the 2D classification. After this step, we retained 1,934,043 particles that exhibited the most consistent and well‐defined features for three‐dimensional (3D) classification. Continuing with the 3D classification, we applied C1 symmetry. This step helped to further refine the particle selection, and we selected 1,300,114 particles for D2 symmetry 3D classification. Subsequently, we re‐extracted particles to bin1 and performed 3D refinement. A soft mask focusing on the center CTPS tetramer is applied in all remaining refinement steps. CTF refinement and Bayesian polishing were applied to improve the map resolution. After these procedures, we obtained a map with a resolution of 2.9 Å. Subsequent quality enhancement of the map was performed using ResolveEM within Phenix, resulting in a final resolution of 2.8 Å.

### Model building and refinement

We employed Model PDB ID 5U3C as the initial model. Initially, a rigid body fitting was conducted using Phenix^40^, followed by manual coordinate adjustments using Coot^41^. Subsequently, real‐space refinement was carried out once again using Phenix.

### Statistical analysis

Results of the CTPS activity assay were analyzed using GraphPad Prism 8^42^ and were shown as means ± SD of three or more independent experiments. The MUSCLE^37^ program was used for sequence alignment.

## AUTHOR CONTRIBUTIONS

**Chenjun Guo**: Conceptualization (lead); formal analysis (equal); investigation (equal); writing—original draft (lead); writing—review and editing (equal). **Zixuan Wang**: Conceptualization (equal); formal analysis (equal); investigation (equal); writing—original draft (supporting); writing—review and editing (supporting). **Ji‐Long Liu**: Conceptualization (equal); funding acquisition (lead); supervision (lead); writing—review and editing (equal).

## ETHICS STATEMENT

No animals or humans were involved in this study.

## CONFLICT OF INTERESTS

The authors declare no conflict of interests.

## Supporting information

Supporting information.

Supporting information.

Supporting information.

Supporting information.

Supporting information.

Supporting information.

Supporting information.

Supporting information.

Supporting information.

Supporting information.

## Data Availability

The structure data accession codes are EMD‐35278 and PDB‐8I9O.

## References

[1] Endrizzi JA , Kim H , Anderson PM , Baldwin EP . Crystal structure of *Escherichia coli* cytidine triphosphate synthetase, a nucleotide‐regulated glutamine amidotransferase/ATP‐dependent amidoligase fusion protein and homologue of anticancer and antiparasitic drug targets. Biochemistry. 2004;43:6447–6463. 15157079 10.1021/bi0496945 PMC2891762

[2] Weng ML , Zalkin H . Structural role for a conserved region in the CTP synthetase glutamine amide transfer domain. J Bacteriol. 1987;169:3023–3028. 3298209 10.1128/jb.169.7.3023-3028.1987 PMC212343

[3] Lieberman I . Enzymatic amination of uridine triphosphate to cytidine triphosphate. J Biol Chem. 1956;222:765–775. 13367044

[4] Ellims PH , Gan TE , Medley G . Cytidine triphosphate synthetase activity in lymphoproliferative disorders. Cancer Res. 1983;43:1432–1435. 6572096

[5] Williams JC , Kizaki H , Weber G , Morris HP . Increased CTP synthetase activity in cancer cells. Nature. 1978;271:71–73. 203856 10.1038/271071a0

[6] Kizaki H , Williams JC , Morris HP , Weber G . Increased cytidine 5′‐triphosphate synthetase activity in rat and human tumors. Cancer Res. 1980;40:3921–3927. 7471043

[7] Mori G , Chiarelli LR , Esposito M , Makarov V , Bellinzoni M , Hartkoorn RC , et al. Thiophenecarboxamide derivatives activated by EthA kill *Mycobacterium tuberculosis* by inhibiting the CTP synthetase PyrG. Chem Biol. 2015;22:917–927. 26097035 10.1016/j.chembiol.2015.05.016 PMC4521081

[8] Hofer A , Steverding D , Chabes A , Brun R , Thelander L . *Trypanosoma brucei* CTP synthetase: a target for the treatment of African sleeping sickness. Proc Natl Acad Sci USA. 2001;98:6412–6416. 11353848 10.1073/pnas.111139498 PMC33482

[9] De Clercq E . Antiviral agents: characteristic activity spectrum depending on the molecular target with which they interact. Adv Virus Res. 1993;42:1–55. 8430518 10.1016/s0065-3527(08)60082-2

[10] McPartland RP , Wang MC , Bloch A , Weinfeld H . Cytidine 5′‐triphosphate synthetase as a target for inhibition by the antitumor agent 3‐deazauridine. Cancer Res. 1974;34:3107–3111. 4472653

[11] Chang Y‐F , Carman GM . CTP synthetase and its role in phospholipid synthesis in the yeast *Saccharomyces cerevisiae* . Prog Lipid Res. 2008;47:333–339. 18439916 10.1016/j.plipres.2008.03.004 PMC2583782

[12] Bearne SL , Guo CJ , Liu JL . GTP‐dependent regulation of CTP synthase: evolving insights into allosteric activation and NH_3_ translocation. Biomolecules. 2022;12:647. 35625575 10.3390/biom12050647 PMC9138612

[13] Habrian C , Chandrasekhara A , Shahrvini B , Hua B , Lee J , Jesinghaus R , et al. Inhibition of *Escherichia coli* CTP synthetase by NADH and other nicotinamides and their mutual interactions with CTP and GTP. Biochemistry. 2016;55:5554–5565. 27571563 10.1021/acs.biochem.6b00383 PMC5584805

[14] Levitzki A , Koshland Jr. DE . Ligand‐induced dimer‐to‐tetramer transformation in cytosine triphosphate synthetase. Biochemistry. 1972;11:247–253. 4550560 10.1021/bi00752a016

[15] Pappas A , Yang WL , Park TS , Carman GM . Nucleotide‐dependent tetramerization of CTP synthetase from *Saccharomyces cerevisiae* . J Biol Chem. 1998;273:15954–15960. 9632643 10.1074/jbc.273.26.15954

[16] Hansen JM , Horowitz A , Lynch EM , Farrell DP , Quispe J , DiMaio F , et al. Cryo‐EM structures of CTP synthase filaments reveal mechanism of pH‐sensitive assembly during budding yeast starvation. eLife. 2021;10:e73368. 34734801 10.7554/eLife.73368 PMC8641951

[17] Lynch EM , DiMattia MA , Albanese S , van Zundert GCP , Hansen JM , Quispe JD , et al. Structural basis for isoform‐specific inhibition of human CTPS1. Proc Natl Acad Sci USA. 2021;118:e2107968118. 34583994 10.1073/pnas.2107968118 PMC8501788

[18] Zhou X , Guo CJ , Chang CC , Zhong J , Hu HH , Lu GM , et al. Structural basis for ligand binding modes of CTP synthase. Proc Natl Acad Sci USA. 2021;118:e2026621118. 34301892 10.1073/pnas.2026621118 PMC8325340

[19] Ingerson‐Mahar M , Briegel A , Werner JN , Jensen GJ , Gitai Z . The metabolic enzyme CTP synthase forms cytoskeletal filaments. Nat Cell Biol. 2010;12:739–746. 20639870 10.1038/ncb2087 PMC3210567

[20] Barry RM , Bitbol A‐F , Lorestani A , Charles EJ , Habrian CH , Hansen JM , et al. Large‐scale filament formation inhibits the activity of CTP synthetase. eLife. 2014;3:e03638. 25030911 10.7554/eLife.03638 PMC4126345

[21] Lynch EM , Hicks DR , Shepherd M , Endrizzi JA , Maker A , Hansen JM , et al. Human CTP synthase filament structure reveals the active enzyme conformation. Nat Struct Mol Biol. 2017;24:507–514. 28459447 10.1038/nsmb.3407 PMC5472220

[22] Zhou X , Guo C‐J , Hu H‐H , Zhong J , Sun Q , Liu D , et al. *Drosophila* CTP synthase can form distinct substrate‐and product‐bound filaments. J Genet Genomics. 2019;46:537–545. 31902586 10.1016/j.jgg.2019.11.006

[23] Lynch EM , Kollman JM . Coupled structural transitions enable highly cooperative regulation of human CTPS2 filaments. Nat Struct Mol Biol. 2020;27:42–48. 31873303 10.1038/s41594-019-0352-5 PMC6954954

[24] Liu J‐L . Intracellular compartmentation of CTP synthase in *Drosophila* . J Genet Genomics. 2010;37:281–296. 20513629 10.1016/S1673-8527(09)60046-1

[25] Noree C , Sato BK , Broyer RM , Wilhelm JE . Identification of novel filament‐forming proteins in *Saccharomyces cerevisiae* and *Drosophila melanogaster* . J Cell Biol. 2010;190:541–551. 20713603 10.1083/jcb.201003001 PMC2928026

[26] Zhang Y , Liu J , Liu JL . The atlas of cytoophidia in *Drosophila* larvae. J Genet Genomics. 2020;47:321–331. 32912804 10.1016/j.jgg.2020.06.004

[27] Liu J , Zhang Y , Zhou Y , Wang QQ , Ding K , Zhao S , et al. Cytoophidia coupling adipose architecture and metabolism. Cell Mol Life Sci. 2022;79:534. 36180607 10.1007/s00018-022-04567-w PMC11802969

[28] Hatse S , De Clercq E , Balzarini J . Role of antimetabolites of purine and pyrimidine nucleotide metabolism in tumor cell differentiation. Biochem Pharmacol. 1999;58:539–555. 10413291 10.1016/s0006-2952(99)00035-0

[29] Fijolek A , Hofer A , Thelander L . Expression, purification, characterization, and in vivo targeting of trypanosome CTP synthetase for treatment of African sleeping sickness. J Biol Chem. 2007;282:11858–11865. 17331943 10.1074/jbc.M611580200

[30] McCluskey GD , Mohamady S , Taylor SD , Bearne SL . Exploring the potent inhibition of CTP synthase by gemcitabine‐5′‐triphosphate. ChemBioChem. 2016;17:2240–2249. 27643605 10.1002/cbic.201600405

[31] McLeod MJ , Tran N , McCluskey GD , Gillis TD , Bearne SL , Holyoak T . A metal‐dependent conformational change provides a structural basis for the inhibition of CTP synthase by gemcitabine‐5′‐triphosphate. Prot Sci. 2023;32:e4648. 10.1002/pro.4648 PMC10182726 37106216

[32] Levitzki A , Koshland DE . Cytidine triphosphate synthetase. Covalent intermediates and mechanisms of action. Biochemistry. 1971;10:3365–3371. 4940761 10.1021/bi00794a008

[33] Long CW , Pardee AB . Cytidine triphosphate synthetase of *Escherichia coli* B. I. purification and kinetics. J Biol Chem. 1967;242:4715–4721. 4862983

[34] Bairoch A , Boeckmann B . The SWISS‐PROT protein sequence data bank. Nucleic Acids Res. 1991;19(Suppl):2247–2249. 2041811 10.1093/nar/19.suppl.2247 PMC331359

[35] Michener CD , Sokal RR . A quantitative approach to a problem in classification. Evolution. 1957;11:130–162.

[36] Tamura K , Stecher G , Kumar S . MEGA11: molecular evolutionary genetics analysis version 11. Mol Biol Evol. 2021;38:3022–3027. 33892491 10.1093/molbev/msab120 PMC8233496

[37] Edgar RC . MUSCLE: multiple sequence alignment with high accuracy and high throughput. Nucleic Acids Res. 2004;32:1792–1797. 15034147 10.1093/nar/gkh340 PMC390337

[38] Mirdita M , Schütze K , Moriwaki Y , Heo L , Ovchinnikov S , Steinegger M . ColabFold: making protein folding accessible to all. Nat Methods. 2022;19:679–682. 35637307 10.1038/s41592-022-01488-1 PMC9184281

[39] Robert X , Gouet P . Deciphering key features in protein structures with the new ENDscript server. Nucleic Acids Res. 2014;42:W320–W324. 24753421 10.1093/nar/gku316 PMC4086106

[40] Adams PD , Grosse‐Kunstleve RW , Hung LW , Ioerger TR , McCoy AJ , Moriarty NW , et al. PHENIX: building new software for automated crystallographic structure determination. Acta Crystallogr D Biol Crystallogr. 2002;58:1948–1954. 12393927 10.1107/s0907444902016657

[41] Emsley P , Lohkamp B , Scott WG , Cowtan K . Features and development of Coot. Acta Crystallogr D Biol Crystallogr. 2010;66:486–501. 20383002 10.1107/S0907444910007493 PMC2852313

[42] Swift ML . GraphPad prism, data analysis, and scientific graphing. J Chem Inf Comput Sci. 1997;37:411–412.

