# Supplementary material for: Filamentation and inhibition of prokaryotic CTP synthase with ligands
Source: mLife. 2024 May 2;3(2):240–50. doi: 10.1002/mlf2.12119 (PMC11211670; doi:10.1002/mlf2.12119)
Supplement: Supplementary file 1 — Supporting information. [file MLF2-3-240-s006.pdf]

Table S1. Cryo-EM data collection and model refinement		
Model	PDB ID 8I9O EMDB-35278	
**Data collection**		
EM equipment	Titan Krios	
Detector	K3 camera	
Magnification	22,500x	
Voltage (kV)	300	
Electron exposure ((e–/Å^2))	50	
Defocus range(μm)	-0.8 to -1.6	
Pixel size(Å)	0.53	
Symmetry imposed	D2	
Number of collected movies	4204	
Initial particle images (no.)	3401539	
Final particle images (no.)	1300114	
**Refinement**		
Composition		
Chains	3	
Atoms	4150 (Hydrogens: 0)	
Residues	Protein: 527 Nucleotide: 1	
Water	0.00	
Ligands	MG: 1	
	CTP: 1	
Bonds (RMSD)		
Length (Å)	0.003 (0)	
Angles (°)	0.518 (0)	
MolProbity score	1.98	
Clash score	5.91	
Ramachandran plot (%)		
Outliers	0.00	
Allowed	3.83	
Favored	96.17	
Rama-Z (Ramachandran plot Z-score, RMSD)		
whole (N = 2216)	1.45 (0.37)	
helix (N = 888)	2.57 (0.35)	
sheet (N = 372)	1.57 (0.51)	
loop (N = 956)	-1.14 (0.40)	
Rotamer outliers (%)	3.37	
C-α outliers (%)	0	
Peptide plane (%)		
Cis proline/general	0.0/0.0	
Twisted proline/general	0.0/0.0	
CaBLAM outliers (%)	0	
ADP (B-factors)		
Iso/Aniso	4150/0	
min/max/mean		
Protein	0.00/91.89/38.92	
Nucleotide	41.53/47.38/43.84	
Ligand	0.00/25.64/14.13	
Water	---	
Occupancy		
Mean	1.00	
occ = 1 (%)	100.00	
0 < occ < 1 (%)	0.00	
occ > 1 (%)	0.00	
Data		
Box		
Lengths (Å)	89.04,65.72,91.16	
Angles (°)	90,90,90	
Supplied Resolution (Å)	2.90	
Resolution Estimates (Å)	Masked	Unmasked
d FSC (half maps; 0.143)	---	---
d 99 (full/half1/half2)	2.7/---/---	2.7/---/---
d model	2.80	2.80
d FSC model (0/0.143/0.5)	2.5/2.6/2.9	2.5/2.6/2.8
Map min/max/mean	-8.90/13.84/0.00	
Model vs. Data		
CC (mask)	0.85	
CC (box)	0.47	
CC (peaks)	0.36	
CC (volume)	0.76	
Mean CC for ligands	0.82	
