# Supplementary figures and images for: Filamentation and inhibition of prokaryotic CTP synthase with ligands
Source: mLife. 2024 May 2;3(2):240–50. doi: 10.1002/mlf2.12119 (PMC11211670; doi:10.1002/mlf2.12119)

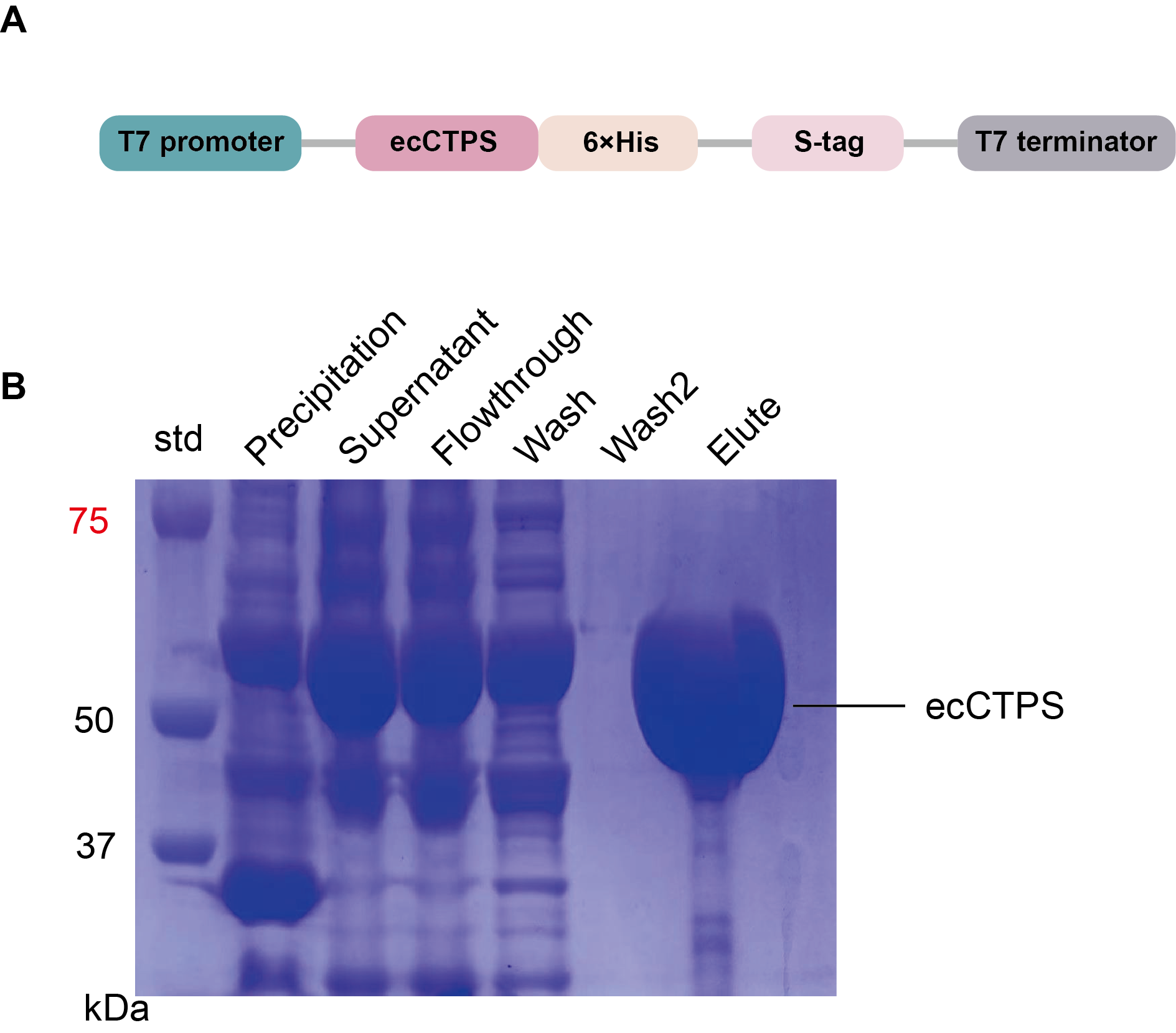

Supplement: Supplementary file 2 — Supporting information. [file MLF2-3-240-s007.png]

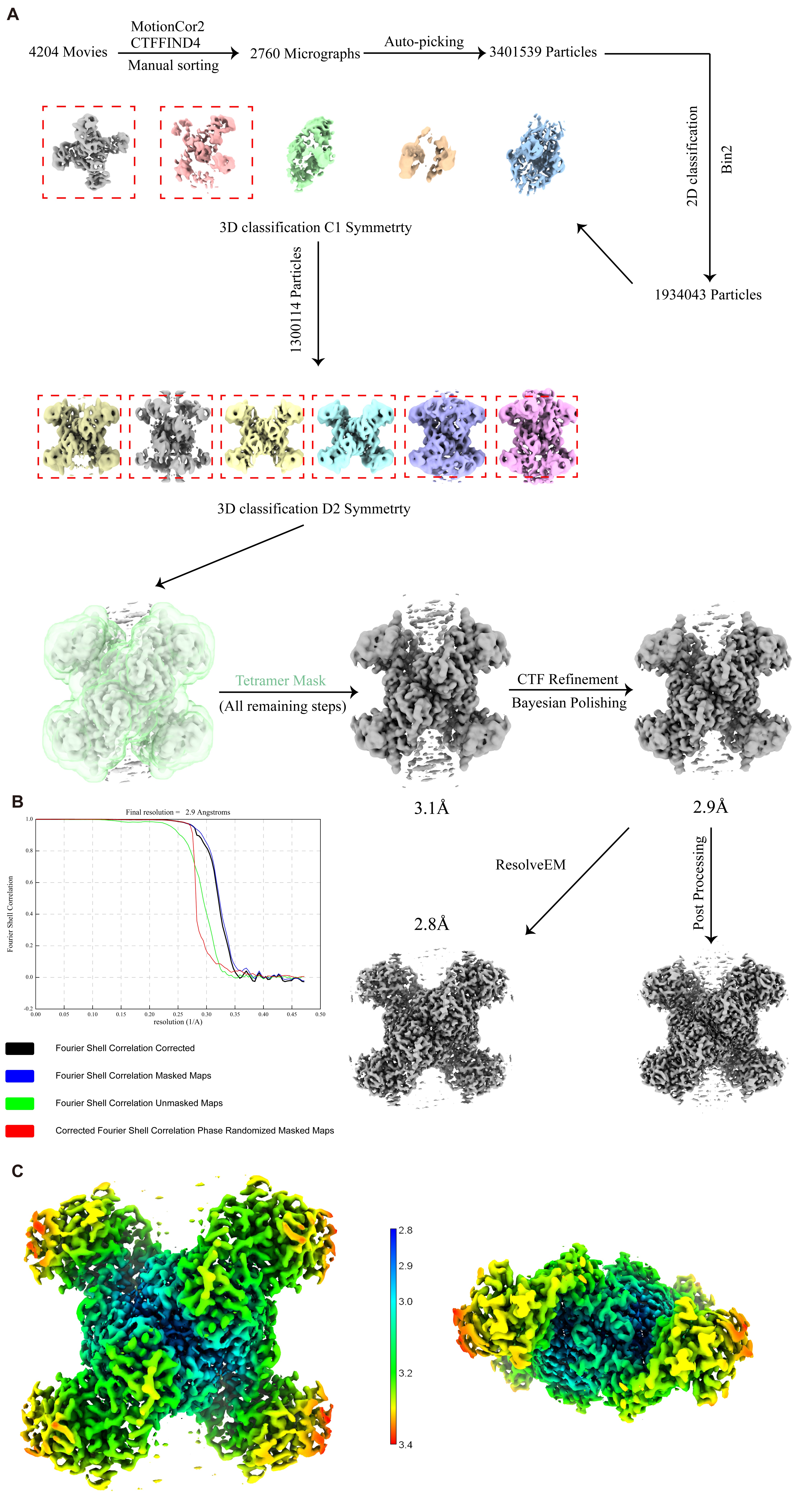

Supplement: Supplementary file 3 — Supporting information. [file MLF2-3-240-s008.jpg]

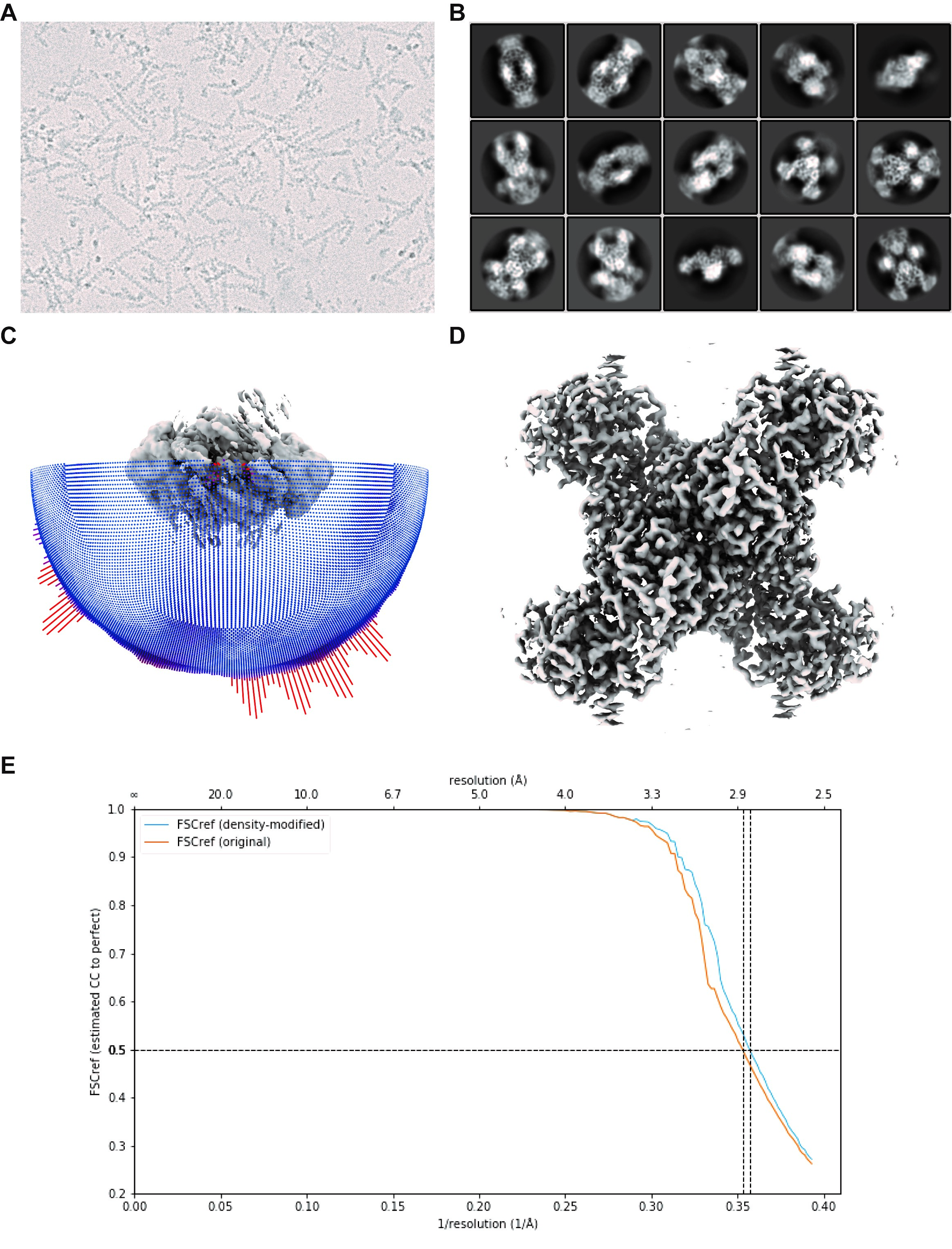

Supplement: Supplementary file 4 — Supporting information. [file MLF2-3-240-s003.jpg]

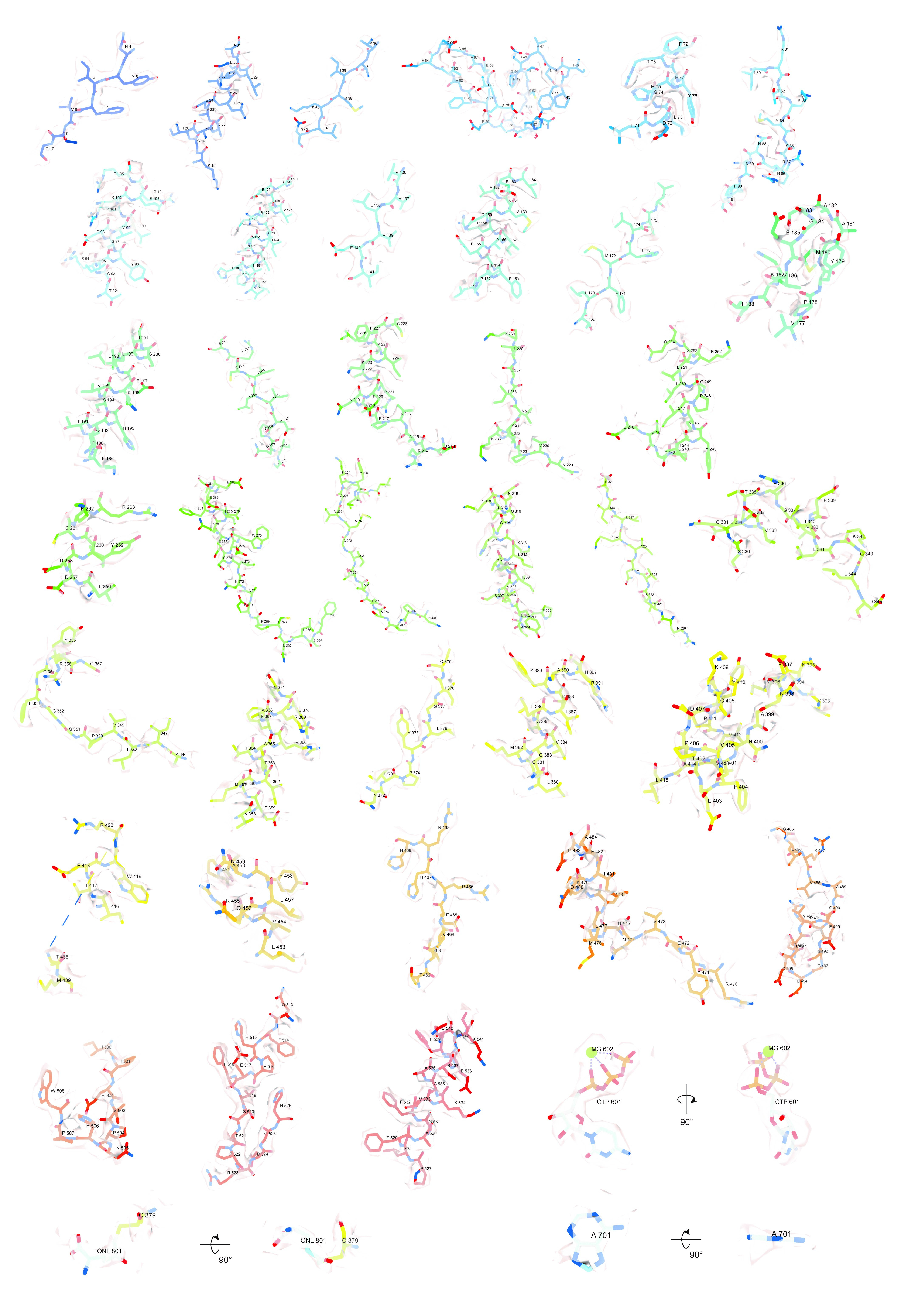

Supplement: Supplementary file 5 — Supporting information. [file MLF2-3-240-s002.jpg]

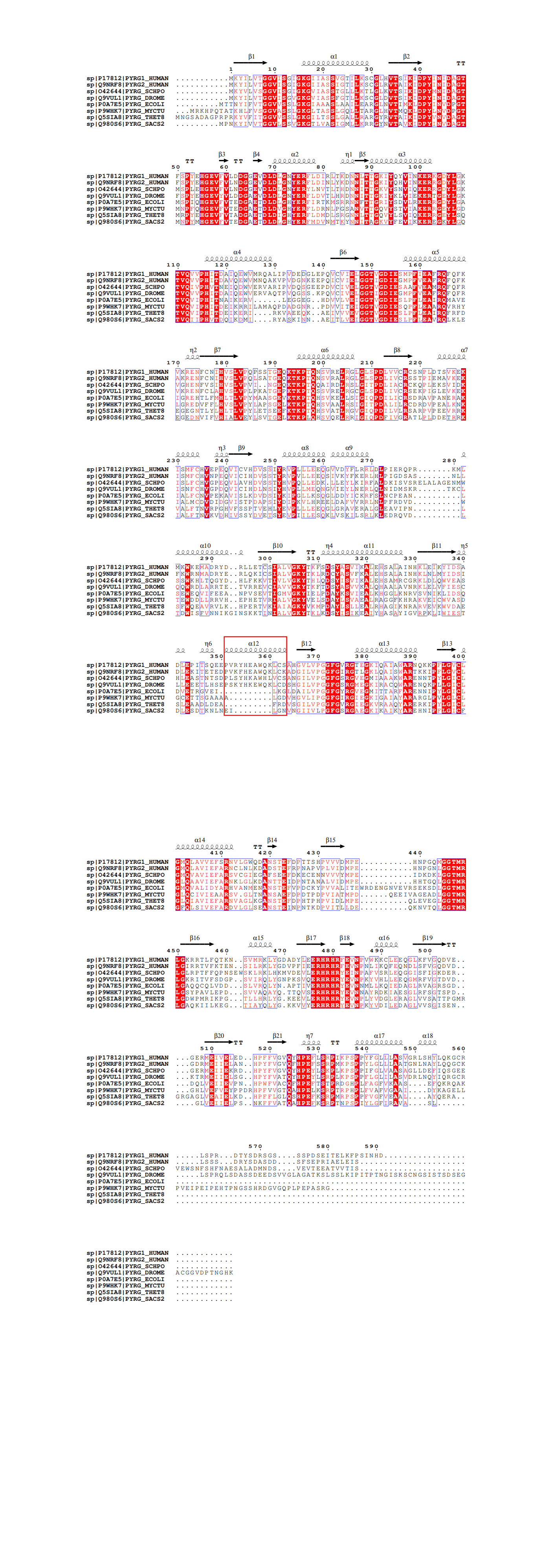

Supplement: Supplementary file 6 — Supporting information. [file MLF2-3-240-s001.png]

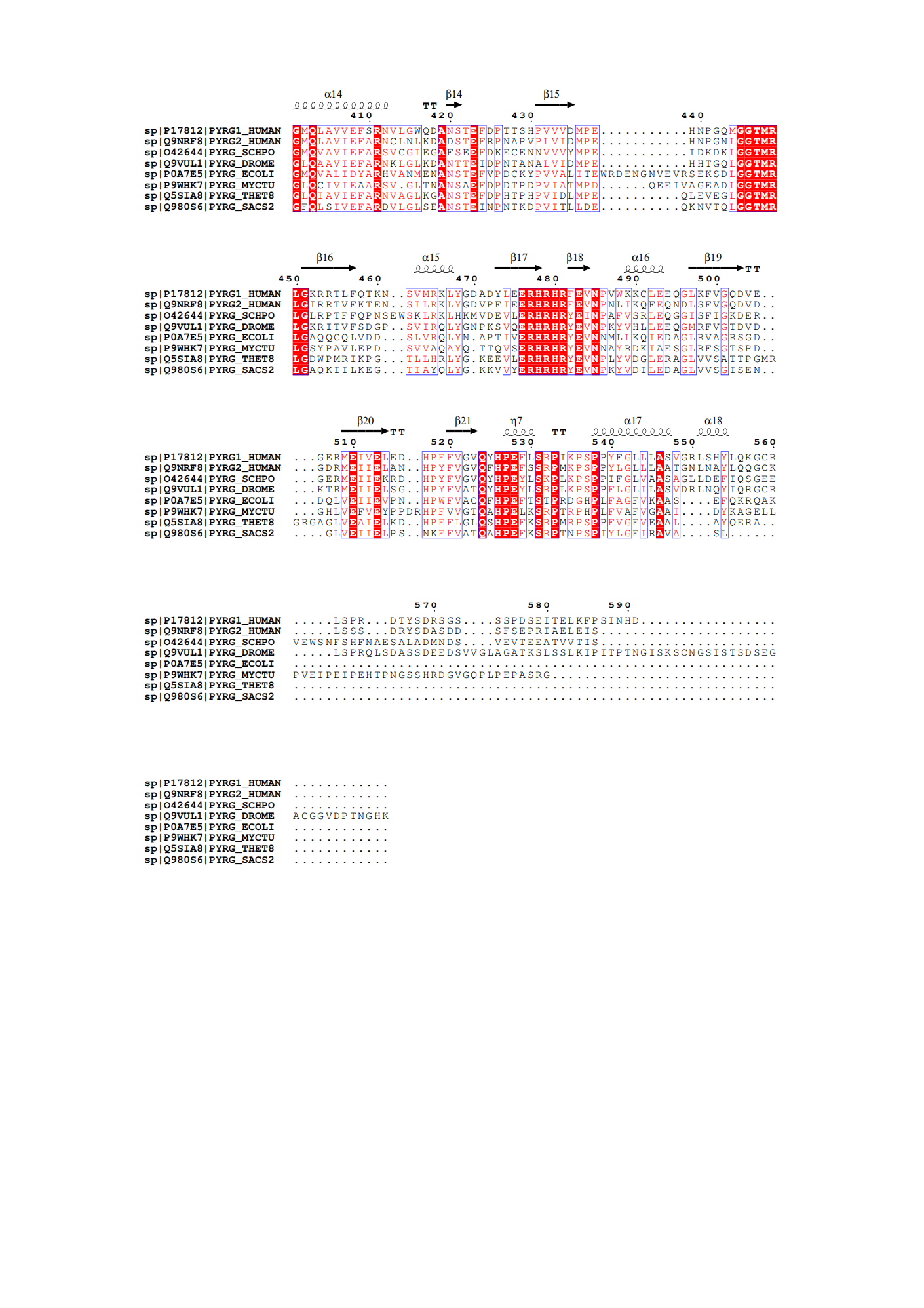

Supplement: Supplementary file 7 — Supporting information. [file MLF2-3-240-s009.png]

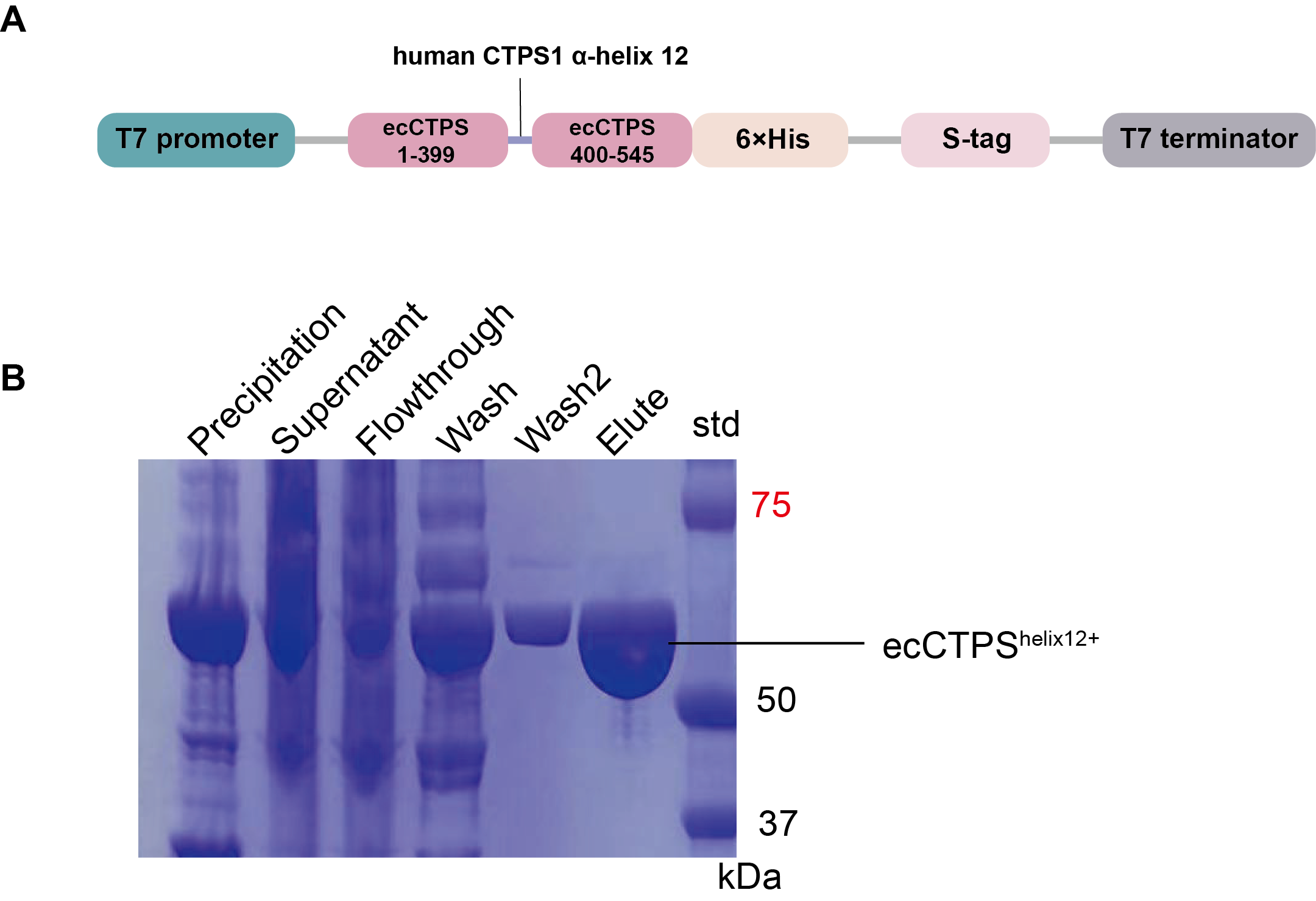

Supplement: Supplementary file 9 — Supporting information. [file MLF2-3-240-s010.png]

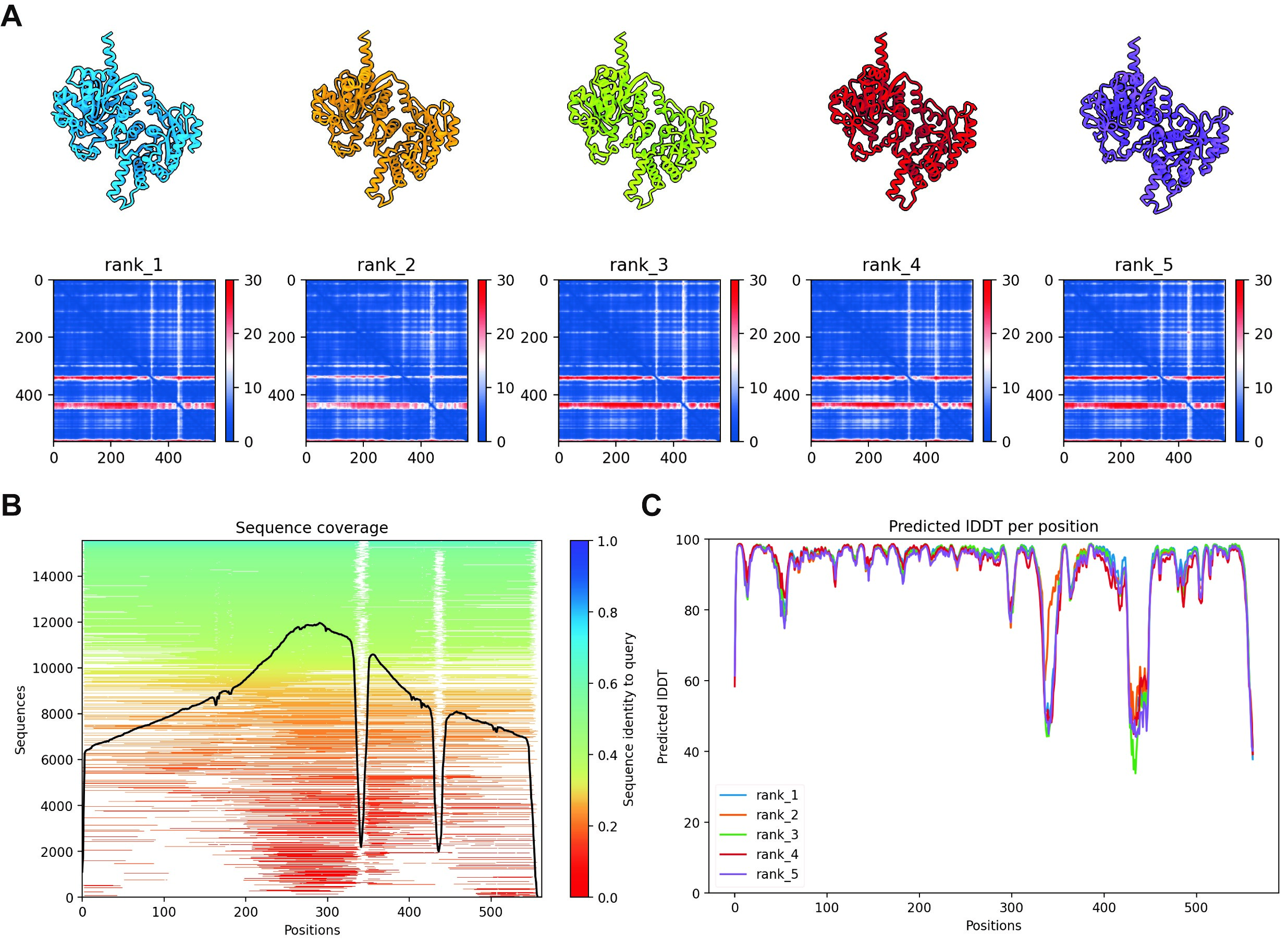

Supplement: Supplementary file 10 — Supporting information. [file MLF2-3-240-s004.jpg]
